# Update on Pyrin Functions and Mechanisms of Familial Mediterranean Fever

**DOI:** 10.3389/fmicb.2016.00456

**Published:** 2016-03-31

**Authors:** Gayane Manukyan, Rustam Aminov

**Affiliations:** ^1^Group of Molecular and Cellular Immunology, Institute of Molecular Biology, National Academy of SciencesYerevan, Armenia; ^2^School of Medicine and Dentistry, University of AberdeenAberdeen, UK

**Keywords:** familial Mediterranean fever, autoinflammation, pyrin, innate immunity, cytoskeleton

## Abstract

Mutations in the *MEFV* gene, which encodes the protein named pyrin (also called marenostrin or TRIM20), are associated with the autoinflammatory disease familial Mediterranean fever (FMF). Recent genetic and immunologic studies uncovered novel functions of pyrin and raised several new questions in relation to FMF pathogenesis. The disease is clinically heterogeneous reflecting the complexity and multiplicity of pyrin functions. The main functions uncovered so far include its involvement in innate immune response such as the inflammasome assemblage and, as a part of the inflammasome, sensing intracellular danger signals, activation of mediators of inflammation, and resolution of inflammation by the autophagy of regulators of innate immunity. Based on these functions, the FMF-associated versions of pyrin confer a heightened sensitivity to a variety of intracellular danger signals and postpone the resolution of innate immune responses. It remains to be demonstrated, however, what kind of selective advantage the heterozygous carriage conferred in the past to be positively selected and maintained in populations from the Mediterranean basin.

## Introduction

Autoinflammatory diseases are a group of genetically determined multisystem disorders caused primarily by the dysfunctions in innate immunity. These rare disorders are characterized by recurrent episodes of generalized inflammation and fever in the absence of infectious or autoimmune causes (McDermott et al., 1999; Brydges and Kastner, 2006). Familial Mediterranean fever (FMF; MIM 294100) is one of the most common and best characterized hereditary autoinflammatory syndromes (Ting et al., 2006; Masters et al., 2009). FMF is an ethnically restricted disease, which predominantly affects people of Mediterranean descent, mainly Armenians, Turks, Arabs and Jews. The carrier rate in these populations is estimated to be as high as 1:5 to 1:7 (Daniels et al., 1995; Gershoni-Baruch et al., 2001; Yilmaz et al., 2001; Sarkisian et al., 2008). Other Mediterranean populations are less affected by the disease, although the number of cases is still substantial (Touitou, 2001; La Regina et al., 2003).

## Genetics of FMF

Familial Mediterranean fever has long being considered as an autosomal recessive disease caused by mutations in the *MEFV* gene, which is composed of 10 exons and encodes a 781 amino acids protein called pyrin or marenostrin or TRIM20 (The French FMF Consortium, 1997; The International FMF Consortium, 1997). According to the INFEVERS database, more than 60 FMF-associated mutations have been identified so far, with the majority of them being extremely rare mutations (http://fmf.igh.cnrs.fr/infevers/). The FMF-associated mutations are predominantly located within exon 10 of the gene, and they primarily result in amino acid substitutions. The phenotypic variability of the disease is thought to be partially associated with particular mutations and allelic heterogeneity (Dewalle et al., 1998; Shohat et al., 1999). The most frequent mutations causing FMF are the five missense mutations: in exon 10 these are M694V, M694I, V726A, and M680I and in exon 2 – E148Q (Touitou, 2001). Among these five mutations, M694V is associated with a more severe form of the disease (Shinar et al., 2000), and E148Q mutation – with a milder form of the disease (Touitou, 2001).

Despite being long considered as an autosomal recessive disorder, there are some cases with the involvement of only one *MEFV* heterozygous mutation and even without any apparent *MEFV* mutation (Booty et al., 2009; Marek-Yagel et al., 2009; Ben-Zvi et al., 2015). Although the genetic defect causing the disease is well-known, a broad spectrum of *MEFV*-associated phenotypes suggest additional genes or immune factors, which may modulate the innate immune response in FMF. A growing number of studies confirm this hypothesis. It has been suggested that major histocompatibility complex class (HLA) I chain-related gene A has a modifier effect on the disease phenotype (Turkcapar et al., 2007). More recently, differential effects of HLA class I and class II alleles on FMF such as subsets of clinical forms and response to colchicines treatment have been demonstrated for the Japanese population (Yasunami et al., 2015).

The high carrier frequency in affected populations suggests the selective advantage conferred by the heterozygous state such as protection against presumptive pathogen(s). Comparative analyses of amino acid substitutions in the ret finger protein (rfp) domain of pyrin among primates and diseased people have demonstrated that some human mutations actually represent the recapitulation to the ancestral amino acid states and these exist as wild type in other species (Schaner et al., 2001). Inspection of the dN/dS ratios performed by the authors revealed the signature of episodic positive selection. Another study of population genetics of FMF involving the sequence analysis of a larger *MEFV* region, from exon 5 to the 3′-UTR, essentially supported the hypothesis of heterozygote advantage/overdominance for *MEFV* mutations (Fumagalli et al., 2009). The meta-analysis of population genetics in FMF suggests that the mutations are not uniformly distributed in various populations and differ in phenotypes, neutrality and other population genetics characteristics (Papadopoulos et al., 2008). The authors identified Jews as the candidate population for founder effects in *MEFV* mutations due to genetic isolation and genetic drift.

Positive selection to maintain the high frequency of the heterozygotes should be sufficiently strong to overcome the negative effects such as the increased morbidity and mortality rates among the homozygotes and compounded heterozygotes (Twig et al., 2014). There have been suggestions that the mutated forms of the protein may confer an increased protection against tuberculosis (Cattan, 2003) or brucellosis (Ross, 2007) but none had direct evidence provided. Other potential effects of the mutation carrier state are thought may be associated with the protection against asthma (Brenner-Ullman et al., 1994), atopy (Sackesen et al., 2004), or allergy (Kalyoncu et al., 2006). But it is not clear then why this protection mechanism against allergic diseases had not emerged worldwide but remained restricted to the existing geographical and ethnical boundaries.

## Pyrin: Structure and Protein–Protein Interaction

*MEFV* encodes the protein called pyrin (also known as marenostrin or TRIM20), which is supposed to play a key role in apoptotic and inflammatory signaling pathways. The protein belongs to the large family of proteins sharing a conserved domain structure with the tripartite motif (TRIM) consisting of an N-terminal RING domain, B-box domain(s) and a C-terminal coiled-coil domain (Weinert et al., 2015). In TRIM20 the RING domain with the ubiquitin ligase activity is replaced by the PYD domain, which belongs to the death domain superfamily (Martinon et al., 2001; Gumucio et al., 2002; Kohl and Gruütter, 2004). It also carries an additional ∼200-amino acid C-terminal B30.2/rfp/PRY/SPRY domain. The majority of the disease-associated mutations are located in the C-terminal B30.2 domain.

During the last decade the functions of pyrin have been the subject of intensive research, and advances in our understanding of its functionality are the result of identification of its interaction with different proteins and oligomers. In particular, the PYD domain, via cognate pyrin domain association, interacts with an adapter protein denoted apoptosis-associated speck-like protein with a caspase recruitment domain (ASC; Richards et al., 2001), and thus participates in the regulation of apoptosis, inflammation, and IL-1β processing (Gumucio et al., 2002). The B30.2/rfp/PRY/SPRY domain-mediated protein interactions indicate the role of this domain as an adaptor module to assemble macromolecular complexes (Perfetto et al., 2013). As a part of the pyrin inflammasome, pyrin also acts as a pattern recognition receptor sensing pathogen modification and inactivation of Rho GTPases (Xu et al., 2014). Paradoxically, pyrin also acts as a component directing the inflammasome components, NLRP1, NLRP3, and pro-caspase 1, to selective autophagic degradation (Kimura et al., 2015). On its own, the dimerization of B30.2 domains by means of the CHS domain appears to be crucial for the recognition of higher order oligomers (Weinert et al., 2015).

## Regulation of Innate Immunity by Pyrin

Since the positional cloning and characterization of the *MEFV* gene in 1997 by two consortia (The French FMF Consortium, 1997; The International FMF Consortium, 1997), numerous hypotheses have been proposed explaining the potential role of the protein encoded in regulation of innate immune responses. Through N-terminal PYD domain pyrin modulates caspase-1 and IL-1β activation exerting proinflammatory (Yu et al., 2006, 2007; Seshadri et al., 2007; Gavrilin et al., 2012) or anti-inflammatory (Chae et al., 2006; Papin et al., 2007; Hesker et al., 2012) regulatory effects, depending on the experimental system employed. In a number of studies the NALP3 inflammasome complex has been implicated in the pathogenesis of FMF (Papin et al., 2007; Omenetti et al., 2014). More recently, homozygous knock-in mice harboring the mouse pyrin protein fused to the human B30.2 domain containing FMF-associated mutations has been shown to secrete the large amounts of IL-1β in a NLRP3-independent manner (Chae et al., 2011) suggesting the formation of an inflammasome that does not include NLRP3. The pro-inflammatory feature of the pyrin function and the formation of the pyrin inflammasome has been confirmed in several later studies (Gavrilin et al., 2012; Mankan et al., 2012; Xu et al., 2014), which suggested that FMF-associated mutations are gain-of-function for pyrin and pyrin itself promotes ASC oligomerization and forms a caspase-1-activating complex. To add even more to the complexity of functions performed by pyrin, a recent study has established that it is involved in the specific autophagic degradation of cytoplasmic regulators of innate immunity thus contributing to the resolution of inflammatory response (Kimura et al., 2015).

## Pyrin and Danger Signals

Initially, the similarity of pyrin with other known transcriptional factors has led to speculations that pyrin may itself be a nuclear factor (The International FMF Consortium, 1997; Centola et al., 2000). It has been shown that the protein product of an alternatively spliced mRNA is able to translocate to the nucleus (Papin et al., 2000). The subsequent studies, however, have demonstrated the cytosolic localization of the full-length pyrin. The first evidence for the interaction of the N-terminal part of pyrin with microtubules and co-localization of pyrin with actin has been obtained by Mansfield and co-workers (Mansfield et al., 2001) suggesting that pyrin is a part of unique cytoskeleton signaling pathway. Subsequently, Waite et al. (2009) confirmed this observation by demonstrating the interaction of pyrin, ASC and actin as well as by identifying new interactions of pyrin with the adaptor protein PSTPIP1, which regulates the cytoskeleton and cell migration. Demonstration of the interaction between the B-box/coiled-coil domains of pyrin with PSTPIP1, the protein that is mutated in PAPA syndrome (Pyogenic Arthritis, Pyoderma gangrenosum, and Acne), confirmed the important role played by pyrin in cytoskeletal signaling pathways (Shoham et al., 2003; Waite et al., 2009). Mutations in PSTPIP1 increase its affinity to pyrin thus suggesting a molecular link between FMF and PAPA syndrome (Shoham et al., 2003).

In a more recent study by Xu et al. (2014) pyrin has been shown to activate caspase-1 in response to Rho GTPases modifying toxins from a number of pathogenic bacteria. They suggested that pyrin recognizes downstream Rho modifications, most likely involving the actin cytoskeleton modifications (Xu et al., 2014). Subsequent studies by Kim et al. (2015) confirmed the link between the aberrant actin depolymerization and inflammasome formation. Moreover, the authors suggested that IL-18 but not IL-1β is implicated in the development of autoinflammatory disease. However, it was shown that pyrin inflammasome is triggered by the lack of actin polymerization using inactivation mutation of the actin-depolymerizing cofactor Wdr1 in mice raised in a gnotobiotic facility (Kim et al., 2015). Thus, the direct participation of pyrin in bacterial sensing has been questioned in this study. Moreover, another interpretation of results by Xu et al. (2014) is that pyrin responds to the modification and inactivation of the host’s proteins by bacterial toxins rather than directly detecting a microbial product (Foley, 2014). In the light of recent findings implicating Rho GTPase in the direct regulation of the pyrin inflammasome the role of the host protein modification as a danger signal is much more prevalent than that of bacterial toxins (Park et al., 2015). Similarly, pertussis toxin with the abolished ADP-ribosyltransferase activity is unable to induce pyrin-dependent inflammasome to cleave pro-IL-1β into the active form (Dumas et al., 2014). Thus the bacterial toxins or components are not the primary danger signals for pyrin inflammasome, there must be modifications of the host proteins in order to be perceived as danger signals.

The relationship between pyrin and cytoskeleton can be additionally confirmed by the therapeutic efficiency of colchicine, an alkaloid with the antimitotic activity, which is recommended and widely used as the first line therapy for the treatment of FMF (Zemer et al., 1986). Moreover, responsiveness to colchicine still has a diagnostic power in FMF. Colchicine exerts various anti-inflammatory effects mostly related to microtubule disruption and depolymerization in a dose-dependent manner (Sackett and Varma, 1993). In a recent study by Taskiran and others the re-organization of actin cytoskeleton in THP-1 cells by colchicine has been described (Taskiran et al., 2012). The mechanisms of its action in FMF have not been completely elucidated and remain under active investigation. It is generally accepted that the therapeutic action of colchicine in FMF is mainly due to the effects on leukocyte migration, signal transduction, and gene expression (Dinarello et al., 1976; Ben-Chetrit et al., 2006; Chae et al., 2008).

Pyrin is primarily expressed in cells of myeloid lineage, mainly polymorphonuclear neutrophils, eosinophils, and monocytes (Centola et al., 2000). Seemingly unprovoked trafficking of these cells to the serosal/synovial membranes is a key event in acute inflammatory attacks of FMF (Ben-Chetrit and Levy, 1998). The regulatory mechanisms implicated in leukocyte migration depend on Ca^2+^ influx and signaling events that result in changes in cytoskeletal organization, such as assembly and disassembly of F-actin, which provide forces necessary for cell migration (Vicente-Manzanares and Sánchez-Madrid, 2004). The regulation of these activities has recently been shown to involve the Rho family of small GTPases, including Rho, Rac, and Cdc42 (Endlich et al., 2001; Provenzano and Keely, 2011). The Rho family of GTPases are the key regulators of a variety of cellular activities including motility, proliferation, apoptosis, and, particularly, of actin cytoskeleton rearrangements (Spiering and Hodgson, 2011). It was shown that Rho activation is sufficient to promote migration of monocytes across endothelial cells (Honing et al., 2004). In our experiments, we have observed a phenomenon of heightened sensitivity of neutrophils from FMF patients toward *in vitro* conditions in the inductor-free media (Manukyan et al., 2013a,b). This suggests that the host-derived stress signals could be responsible for the activation of these cells. Similar results with the use of monocytes have been obtained later by another group (Sugiyama et al., 2014). Experiments *in vitro* unavoidably impose mechanical forces on cells that may affect cytoskeletal structure and modulate cellular behavior (Wang et al., 1993; Matthews et al., 2006). Thus, the routine experimental procedures involving external mechanical forces applied to cells may lead to the generation of danger signals that are sensed by the pyrin inflammasome. Recent investigations showing the pyrin inflammasome activation in response to actin modifications (Xu et al., 2014; Kim et al., 2015), together with our results suggesting the excessive activation of neutrophils from FMF patients in *ex vivo* experiments (Manukyan et al., 2013a,b), warrants further studies of pyrin-cytoskeleton interactions, especially in the case of FMF.

## Clinical Aspects

Clinically FMF divided into two phenotypes, types 1 and 2 (Shohat and Halpern, 2011). Type 1 is characterized by recurrent episodes of fever and polyserositis. Fever is the main symptom of the disease which is present in 95% of acute inflammation episodes, with the body temperature reaching usually above 38°C (Sohar et al., 1967). Fever is accompanied by sterile peritonitis, synovitis, pleuritis, and rarely by pericarditis and erysipelas-like skin lesions (Shohat and Halpern, 2011). A typical attack lasts 12 h to 2–3 days and resolves spontaneously. The frequency, duration, intensity, and symptoms experienced during attacks are highly variable between patients. During attack-free period patients are asymptomatic. About 50% of the patients are reported to have prodromal symptoms (Lidar et al., 2006). The first clinical presentation of type 2 FMF is amyloidosis, in the absence of other clinical symptoms (Shohat and Halpern, 2011).

The manifestation of the disease is diverse, ranging from asymptomatic to the potentially life-threatening states. The main and potentially lethal complication of the disease is secondary (AA) amyloidosis, which usually affects the kidneys. Amyloidosis is the result of tissue deposition of amyloid, which is a proteolytic cleavage product of the acute phase reactant serum amyloid A (SAA; Sohar et al., 1967). Overproduction of SAA leads to the extracellular accumulation of fibrillar protein and the development of amyloidosis (van der Hilst et al., 2005). Presently the administration of colchicine (1–3 mg/day) is the preferred treatment for FMF allowing extended periods of remission as well as preventing amyloidosis in the majority of FMF patients (Pras, 1998). Since 5–10% of the patients are colchicine resistant or intolerant to the drug, the efforts toward developing alternative treatment were undertaken. As an alternative approach, specific anti-cytokine therapies, such as IL-1 receptor and tumor necrosis factor antagonists were tested with a limited number of patients (Nakamura et al., 2007; Ben-Zvi and Livneh, 2014). Although, genotyping of the *MEFV* mutations is the preferred method in FMF diagnostics, it is confirmatory to the clinical diagnosis. Presently, there are no definitive biochemical markers that could serve as diagnostic ones. Usually FMF attacks are accompanied by a non-specific increase in acute phase reactants such as C-reactive protein, fibrinogen, and serum amyloid A as well as by the increased white blood cell count with low-grade neutrophilia. The diagnosis of FMF remains clinical, since mutations have reduced penetrance and cannot always be identified on both alleles (Tunca et al., 2002). Clinical manifestations of FMF may overlap with the phenotypes of autoinflammatory disorders and, to a certain degree, with autoimmune diseases, which may potentially complicate diagnosis of FMF. One distinguishing feature of autoinflammatory syndroms is responsiveness to IL-1β blocking therapy, although there are exceptions (Bodar et al., 2005; Arostegui et al., 2007; Kuijk et al., 2007; Gattorno et al., 2008).

Several studies have demonstrated association of *MEFV* mutations with different inflammatory pathologies, such as systemic onset juvenile idiopathic arthritis (Uslu et al., 2010), inflammatory bowel disease (Akyuz et al., 2013), Behçet’s disease (Atagunduz et al., 2003), ulcerative colitis (Giaglis et al., 2006), Fibromyalgia syndrome (Feng et al., 2009), rheumatoid arthritis (Rabinovich et al., 2005), ankylosing spondylitis (Akkoc et al., 2010), and others. Other pathologies, which are associated with mutations in the *MEFV* gene, are two vasculitis types, Henoch-Schonlein Purpura (Gershoni-Baruch et al., 2003; Tunca et al., 2005) and polyarteritis nodosa (Tunca et al., 2005; Aksu and Keser, 2011). Clinical manifestations of these vasculitis types, which accompany FMF, might be considered as a clinical manifestation of the latter. The diseases listed represent multi-factorial immunological disorders with a marked involvement of the inflammatory component. A growing number of similar cases, which are reported to be associated with the *MEFV* gene, suggest that pyrin is a key regulatory element of the innate immune responses and can affect inflammatory processes during these disorders.

## Concluding Remarks

Understanding the role of pyrin in innate immunity has progressed rapidly in recent years, uncovering its numerous functions in the cell from the formation of several supramolecular structures and inflammasome assembly, to sensing various intracellular danger signals, to mounting the innate immune responses, and to the resolution of inflammation (**Table 1**). The evidences for positive selection of mutations in the *MEFV* gene are given in several works cited in this review, and they have a strong support from the formal analytical approaches of population genetics. What remains unclear, however, why the mutations have been selected and maintained in the Mediterranean populations and what are the mechanistic explanations for the advantage at the biochemical level? In particular, we know well about the negative effects of the homozygous or compounded heterozygous mutant allele combination, which result in a higher morbidity/mortality rate. At the cellular level this genetics presumably results in the impaired assembly of pyrin inflammasomes, in the launch of excessive and extended inflammatory responses, and in a less efficient resolution of inflammation. Given the multiple functions of pyrin and the expected pleiotropic effects of mutations in the *MEFV* gene, the significance and contribution of each of these functions are difficult to ascertain in terms of clinical presentation in pathology or the heterozygote advantage at the phenotypic level. All we know at the clinical level is that the homozygous or compounded heterozygous state results in the enhanced and extended inflammatory response to some of the innocuous factors that are tolerated well and handled efficiently by the normal immune system. The diseased state is certainly disadvantageous (although it cannot be excluded that certain conditions in the past have been conducive to select such traits) but we don’t know about the possible advantages conferred by the heterozygote state. Investigation of this previously ignored group could potentially reveal the characteristics and traits that were responsible for the selective advantage and maintenance of these mutations in the Mediterranean populations.

**Table 1:** Structural and functional roles of pyrin in cellular processes.

Role	Reference
Inflammasome assembly	Chae et al., 2011; Perfetto et al., 2013; Vajjhala et al., 2014
Sensing intracellular danger signals by the inflammasome (aberrant actin depolymerisation, protein modification by bacterial toxins)	Dumas et al., 2014; Xu et al., 2014; Kim et al., 2015
Activation of mediators of inflammation by the inflammasome (IL-1β, IL-18)	Chae et al., 2011; Kim et al., 2015
Pyrin-cytoskeloton interactions	Mansfield et al., 2001; Shoham et al., 2003; Waite et al., 2009
Apoptosis	Martinon et al., 2001; Richards et al., 2001; Gumucio et al., 2002
Autophagy of innate immunity regulators	Mandell et al., 2014; Kimura et al., 2015


## Author Contributions

All authors listed, have made substantial, direct and intellectual contribution to the work, and approved it for publication.

## Conflict of Interest Statement

The authors declare that the research was conducted in the absence of any commercial or financial relationships that could be construed as a potential conflict of interest.
